# Challenging the Diagnosis of Cannabis-Induced Psychosis: A Clinical Perspective on Contaminant-Driven Psychosis in India

**DOI:** 10.1192/j.eurpsy.2026.11108

**Published:** 2026-07-31

**Authors:** O. Rashid

**Affiliations:** 1 Sant Parmanand Hospital Civil Lines, New Delhi, India; 2 Clinical Psychiatry, University of Southwales, Southwales, Cardiff, United Kingdom

## Abstract

**Introduction:**

In India’s context of an unregulated drug supply, psychosis is often attributed to cannabis based on patient history alone. However, the widespread contamination of substances with synthetic cannabinoids, opioids, and novel psychoactive substances (NPS) precipitates severe and atypical psychotic presentations that are frequently misdiagnosed as Cannabis-Induced Psychosis (CIP).

**Objectives:**

- To highlight recurring clinical patterns where features of psychosis are inconsistent with classic CIP, suggesting contamination, and to advocate for greater clinical vigilance and refined diagnostic practices.

**Methods:**

This clinical perspective draws upon experience from multiple centers across India. Illustrative cases were selected to demonstrate prototypical examples where presentation, evolution, and treatment response deviated significantly from expected patterns of phytocannabinoid-induced psychosis, suggesting probable synthetic or polysubstance contamination.

**Results:**

Common atypical features included: (i) extremely rapid onset of psychosis (< 30 minutes); (ii) prominent sedation or catatonic features; (iii) autonomic instability (e.g., tachycardia, hypertension); and (iv) poor response to conventional antipsychotic therapy. These patterns consistently suggested exposure to non-cannabis substances, supporting the need to re-evaluate reliance on patient-reported substance use.

**Conclusions:**

Routine attribution of psychosis to cannabis without toxicological confirmation risks widespread misdiagnosis. Clinicians should consider “contaminant-induced psychosis” in atypical cases and advocate for advanced screening where feasible. A shift in diagnostic mindset is urgently needed to align clinical practice with the reality of the modern illicit drug supply.

**Disclosure of Interest:**

None Declared

